# ER network homeostasis is critical for plant endosome streaming and endocytosis

**DOI:** 10.1038/celldisc.2015.33

**Published:** 2015-11-17

**Authors:** Giovanni Stefano, Luciana Renna, YaShiuan Lai, Erin Slabaugh, Nicole Mannino, Rafael A Buono, Marisa S Otegui, Federica Brandizzi

**Affiliations:** 1 MSU-DOE Plant Research Lab and Plant Biology Department, Michigan State University, East Lansing, MI , USA; 2 Department of Botany, University of Wisconsin-Madison, Madison, WI, USA; 3 R.M. Bock Laboratories Cell and Molecular Biology, University of Wisconsin-Madison, Madison, WI, USA; 4 Department of Genetics, University of Wisconsin-Madison, Madison, WI, USA

**Keywords:** Arabidopsis, endocytosis, endoplasmic reticulum, organelle streaming

## Abstract

Eukaryotic cells internalize cargo at the plasma membrane via endocytosis, a vital process that is accomplished through a complex network of endosomal organelles. In mammalian cells, the ER is in close association with endosomes and regulates their fission. Nonetheless, the physiological role of such interaction on endocytosis is yet unexplored. Here, we probed the existence of ER–endosome association in plant cells and assayed its physiological role in endocytosis. Through live-cell imaging and electron microscopy studies, we established that endosomes are extensively associated with the plant ER, supporting conservation of interaction between heterotypic organelles in evolutionarily distant kingdoms. Furthermore, by analyzing ER–endosome dynamics in genetic backgrounds with defects in ER structure and movement, we also established that the ER network integrity is necessary for homeostasis of the distribution and streaming of various endosome populations as well as for efficient endocytosis. These results support a novel model that endocytosis homeostasis depends on a spatiotemporal control of the endosome dynamics dictated by the ER membrane network.

## Introduction

Endocytosis is an essential process in eukaryotes. It mediates the recycling of membranes, cell surface receptors and ion channels, and is crucially important for the control of the lipid and protein composition of the plasma membrane. Through regulation of either recycling or degradation of plasma membrane receptors, this transport step also contributes to the perception of external stimuli [1, 2]. Endocytosis of cargo is thought to be vesicle mediated and leads to a class of organelles loosely defined as endosomes. These are membranous compartments that function as critical sorting stations for membrane traffic, protein targeting and maturation. The early endosomes (EEs) are the first to receive endocytic cargo [3]. In plant cells, the Trans-Golgi Network (TGN) is the compartment where endocytic cargo accumulates first, and is thus regarded as the plant EE equivalent. It is widely believed that secretory and endocytic routes merge at the TGN/EE, and that endocytic cargo is either recycled back to the plasma membrane or sorted to the vacuole for degradation. The latter pathway passes via the multivesicular bodies (MVBs) or late endosomes (LEs)/prevacuolar compartments and late prevacuolar compartments [4, 5].

In plant cells, intracellular movement of organelles, including endosomes, relies largely on actin [6]. This is in contrast with mammalian cells where endosome movement depends on microtubules. Mammalian endosomes associate with endoplasmic reticulum (ER) tubules, which can wrap around them and regulate their fission [7]. Although it is yet unknown whether the ER–endosome association may have a role in endosomal function in protein traffic, these findings underscore a role of the ER in endosome biogenesis. In plant cells, it is yet unknown whether endosomes associate with the ER and whether the ER may exert a role in endosome biogenesis as well as endosome-driven processes related to membrane traffic homeostasis.

In plant cells, the ER can be found in association with the membranes of other organelles through points of contact that have been seen in electron microscopy [8] and live-cell imaging analyses [9]. However, the physiological relevance of such connections is unknown. Recent findings indicate that connection between the ER and other organelles is significant for organelle streaming, which in plant cells is markedly conspicuous. Although organelle streaming in plant cells depends predominantly on actin and associated myosin motors [10–13], it has been recently shown that reduced membrane fluidity and disruption of the ER network impedes the movement of other organelles [14]. It was postulated that this effect may be linked to alteration of the homeostasis of the contact points between ER and other organelles [14]. Intriguingly, the relevance of organelle movement in the context of organelle function is still an open question. For example, ER–Golgi membrane traffic can take place when the movement of Golgi stacks is inhibited by chemical disruption of actin [15]. However, whether ER–Golgi membrane traffic may be more efficient in intact cells compared with actin-depleted cells is yet to be established. Similarly, it is yet unknown if endosome streaming has a role in endosome-dependent processes, such as endocytosis.

In this work, we addressed the fundamental questions on whether the ER is in close association with endosomes in plant cells, and whether a functional correlation exists between organelle streaming and function. Through live-cell imaging and electron tomography analyses in the plant model species *Arabidopsis thaliana*, we established that endosomes entertain a close association with the ER in plant cells. Using established genetic models with disrupted ER network integrity, we also determined that the ER network is critical for maintaining homeostasis of the spatial distribution and dynamics of endosomes. Finally, using time-course experiments to trace internalization of the fluorescent endocytic probe, FM4-64, as well as analyses of auxin accumulation and distribution of auxin transporters, which obtain a polarized subcellular localization at the plasma membrane through selective endocytosis, we established that defects in the ER network integrity negatively influence endocytosis. Taken together, our results not only support that a close association of the ER with endosomes is conserved among eukaryotic kingdoms, they also offer new perspectives in the significance of organelle movement in plant cells by demonstrating a functional relationship between the dynamics of the ER and the role of endosomes in endocytic traffic.

## Results

### In plant cells, the ER and endosomes are spatially and temporally associated

**Figure 1:**
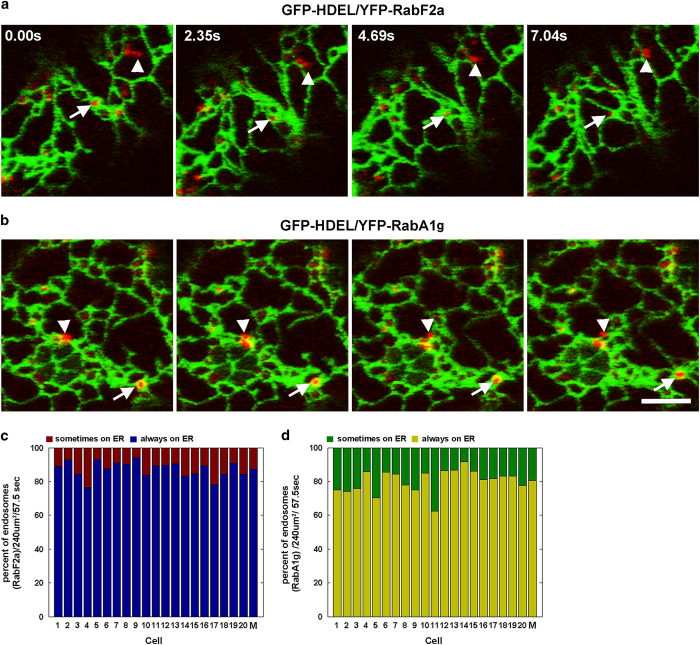
ER and endosomes are associated in plant cells. (**a**, **b**) Time-lapse microscopy in the cortical region of a *Nicotiana tabacum* leaf epidermal cells expressing GFP-HDEL (ER lumen) and either YFP-RabF2a (late endosome/MVBs) or YFP-RabA1g (post-TGN/EE late endosome/pre-vacuolar compartment) reveals that the majority of the endosomes are associated with the ER membranes over time (arrow) or only for a limited time (arrowhead). (**c**, **d**) Quantification of the ER–endosome association over time in individual cells (*N*=20) co-expressing GFP-HDEL and either YFP-RabF2a or YFP-RabA1g. The graph represents the percentage of endosomes that are continuously associated with the ER (always on ER) or are associated only for a limited number of frames (sometimes on ER) during the entire time-lapse sequence (57.5 s). The association was estimated for a total of 689 of RabF2a-labeled endosomes and 1165 RabA1g-labeled endosomes. The average of the percentages estimated for the 20 cells is also presented (M). Scale bars, 5 μm. ER, endoplasmic reticulum; MVBs, multivesicular bodies.

To establish the spatial organization of endosomes in relation with the ER in plant cells, we performed time-course imaging analyses of leaf epidermal cells co-expressing the inert ER lumen marker (GFP-HDEL) [16] and fluorescent protein-tagged RabF2a or RabA1g. RabF2a is a marker of late endosomes/MVBs; RabA1g is a marker of post-TGN/EEs [17, 18]. Using dual-color live-cell imaging, we observed that despite the continuous remodeling of the tubules and cisternal ER, the populations of endosomes labeled by the two Rab-fusion proteins appeared in extensive association with the ER network (Figures 1a and b, Supplementary Movie S1). Specifically, during time-lapse acquisitions we observed that the majority of the endosomes were closely associated with the ER network, while a small subpopulation of endosomes moved between ER tubules. To quantify the degree of association of the endosomes with ER over time, we followed the same approach adopted earlier in mammalian cells to track individual endosomes with respect to the ER network using live-cell imaging of cells co-expressing endosome and ER markers [7]. We tracked the movement of RabF2a- and RabA1g-labeled endosomes with respect to ER tubules for about 1 min. We verified that continuous tracking with the ER was maintained for over 85% of RabF2a-positive endosomes and for over 75% of the RabA1g-positive endosomes. Within the same time frame, the remaining endosomes were found in non-continuous association with the ER network (Figures 1c and d). These data indicate that the majority of the endosome populations visualized with RabF2a and RabA1g are extensively associated with the ER over time.

**Figure 2:**
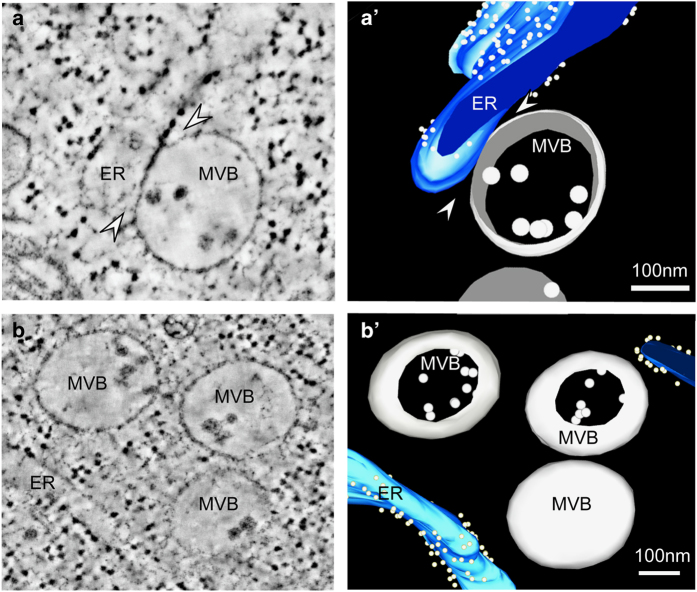
ER–endosome association revealed by electron tomography. Electron tomography slices (**a**, **b**) and corresponding three-dimensional reconstructions (**a**′, **b**′) of *A. thaliana* root epidermal cells showing a close association between the ER membrane and multivesicular endosomes (MVBs; arrowheads). Scale bars, 100 nm. ER, endoplasmic reticulum.

The association between ER and endosomes was further investigated by electron tomography (Figure 2). We obtained dual-axis tomograms of high-pressure frozen/freeze substituted Arabidopsis root epidermal cells and identified MVBs in close proximity to ER membranes. We analyzed 10 tomograms (300-nm-thick) containing 15 MVBs. Physical ER–MVB contact was observed in two cases (Figures 2a and a′), whereas, other MVBs were just at a close distance with the ER (Figures 2b and b′). These results not only support the live-cell imaging observations that endosomes can be found close to the ER but also demonstrate that some of the endosomes may be in physical contact with the ER.

Together, our cell imaging analyses support that the plant ER entertains dynamic associations with the endosomes similar to the mammalian ER [7], underscoring unexpected but significant similarities between the spatial and temporal behavior of endosomal organelles and ER that are conserved across multicellular kingdoms.

### Disruption of ER network integrity affects endosome streaming

**Figure 3:**
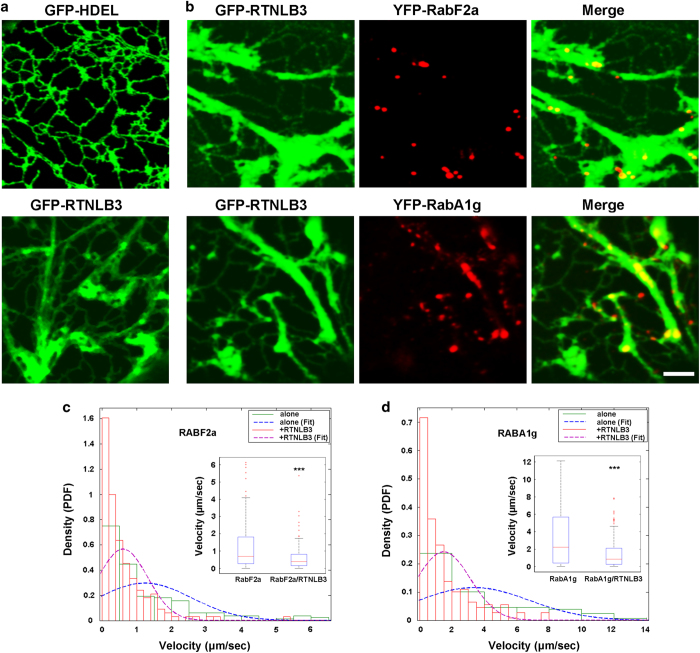
Disruption of ER network integrity compromises endosome velocity. (**a**, **b**) GFP-HDEL, GFP-RTNLB3, YFP-RabF2a and YFP-RabA1g were transiently expressed in tobacco leaf epidermal cells. GFP-HDEL reveals a typical ER network with tubules and cisternae; however, expression of GFP-RTNLB3 causes deformation of the ER network. Scale bars, 5 μm. (**c**, **d**) Streaming velocity measurements of 165 endosome organelles labeled by YFP-RabF2a (**c**) and 218 endosomes labeled by YFP-RabA1g (**d**) in tobacco epidermal cells in the absence (solid green lines) or in the presence of overexpressed GFP-RTNLB3 (solid red lines) were calculated from kymographs generated in ImageJ (v1.49 h). Endosome movement is expressed as histograms and probability density function (PDF) of the velocity distributions, which were fitted (blue and magenta dashed lines) using distribution tool (Matlab). Inset in **c** and **d** display the average velocity of the relative endosomes. Statistical analysis was performed using Student’s *t*-test (***P<0.0001). ER, endoplasmic reticulum.

To establish whether endosome dynamics could be influenced by ER network integrity, we used the known ER membrane-modifier reticulon B3 (RTNLB3), which causes extensive remodeling of ER tubules when overexpressed [19, 20]. Reticulons are a class of membrane proteins that facilitate membrane curvature [21]. In addition to ER network reshaping (Figure 3a), we determined that overexpression of RTNLB3 reduces the diffusion of ER membrane proteins (Supplementary Figure S1). On the basis of observed association of the ER with endosomes (Figures 1 and 2) and the modifying effect of RTNLB3 overexpression on the ER network, we predicted the occurrence of alterations in the dynamics of endosomes in cells overexpressing RTNLB3. To test this, we performed single-organelle streaming measurements in cells expressing the Rab markers alone or in combination with GFP-RTNLB3 (Figures 3b–d). Live-cell imaging analyses of 165 RabF2a-positive endosomes and 218 RabA1g-positive endosomes revealed that the velocity of endosomal streaming was significantly reduced in cells co-expressing GFP-RTNLB3 compared with cells expressing the Rab markers alone. Specifically, kymograph analyses [22] revealed that the average velocities of RabF2a-positive endosomes (1.228±0.105 μm s^−1^) were significantly reduced in cells overexpressing GFP-RTNLB3 (0.605±0.055 μm s^−1^; *P<*0.0001; Figure 3c). A similar trend was observed for the RabA1g-positive endosome population, as demonstrated by the evidence that the endosome velocity (3.356±0.234 μm s^−1^) was significantly reduced when GFP-RTNLB3 was co-expressed (1.482±0.112 μm s^−1^; *P<*0.0001; Figure 3d). Intriguingly, these data indicate that the streaming velocity of RabF2a-positive endosomes is lower than that of RabA1g-positive endosomes. Indeed, colocalization analyses with the two Rab markers showed that the localization of the two endosome markers overlaps only partially (Supplementary Figure S2). Nonetheless, overexpression of the ER-membrane modifier GFP-RTNLB3 led to a similar reduction of streaming (~50%; Figures 3c and d) of both Rab-endosomal populations compared with cells with unmodified ER (Figures 3c and d). On the basis of the results that streaming levels of both populations of endosomes are similarly disrupted by overexpression of the same ER-modifying protein, we conclude that the dynamics of these endosomal populations is largely influenced by homeostasis of the ER membrane network.

**Figure 4:**
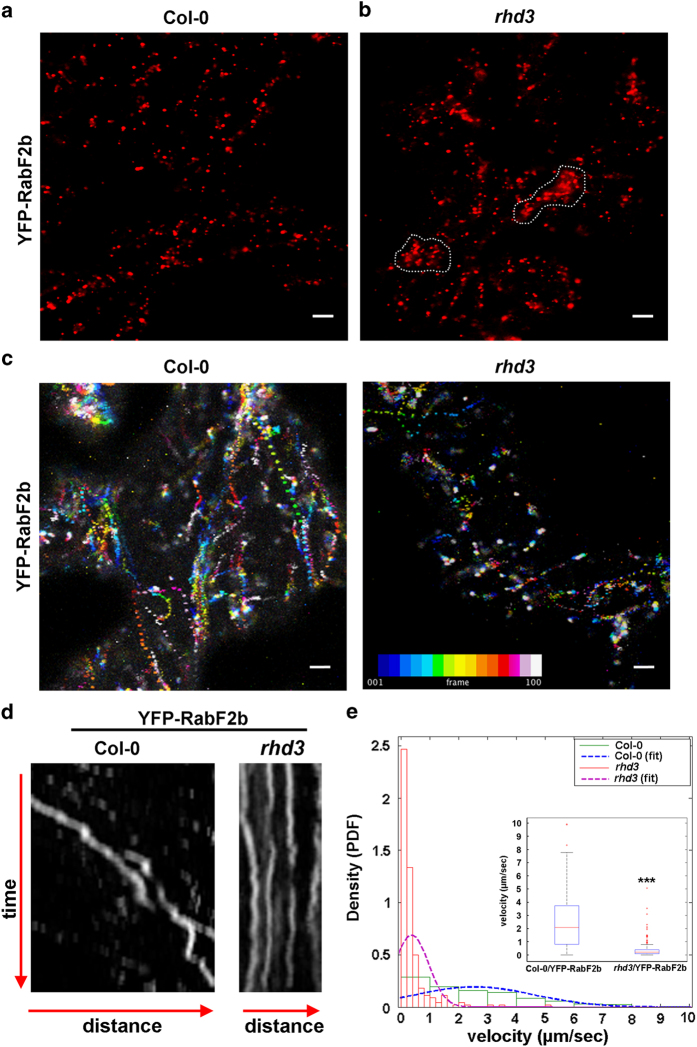
Spatiotemporal distribution of endosome is compromised in *rhd3*. (**a**, **b**) Confocal images of cotyledon epidermal cells in wild type (**a**; Col-0) and *rhd3* (**b**) (*rhd3*) expressing the endosome marker YFP-RabF2b, note the clustering of endosomes in *rhd3* (circled by dotted line). (**c**) Intracellular streaming of endosome organelles in Col-0 and *rhd3* mutant presented as super-imposed frames of a time-lapse sequence (25 s). In each frame, organelles have been assigned a color, with dark red representing the position of organelles in initial frames and white indicating the position of the organelles in the last frame. Intermediated colors are presented at the bottom of the panel. Reduced streaming of endosomes in *rhd3* is evidenced by local superimposition of colors compared with wild type. (**d**). Representative kymographs for Col-0/YFP-RabF2b and *rhd3*/YFP-RabF2b. (**e**) Endosome movement of 221 YFP-RabF2b-labeled endosomes in Col-0 (red) and *rhd3* (green) is expressed as histograms and probability density function (PDF) of the velocity distributions, which were fitted (blue and magenta dashed lines) using distribution tool (Matlab). Statistical analysis was performed using Student’s *t*-test (***P<0.0001). The inset plot shows the average velocity for endosomes in wild type and mutant cells. Scale bars, 5 μm.

To test whether the effect on the spatial distribution and dynamics of the endosomes could be a general phenotype linked to ER defects, we followed the distribution of endosomes in an Arabidopsis knockout mutant of *ROOT HAIR DEFECTIVE 3* (*rhd3*), a critical ER-membrane associated dynamin-like protein that shapes the ER network [23], possibly through mediation of homotypic fusion [24, 25] of ER membranes. Compared with wild-type cells, in *rhd3*-depleted cells the ER network is arranged primarily into clusters of long unbranched tubules (Supplementary Figure S3) and the diffusion of proteins in the ER membrane is reduced [14, 23, 25, 26]. We hypothesized that if the ER integrity exerts a general effect on the spatial and dynamic organization of endosomes, as supported by the experiments with RTNLB3 (Figure 3), then the distribution and streaming velocity of these organelles would be also different in *rhd3* compared with wild type. To test this, we analyzed the distribution of an additional endosome marker, RabF2b, which labels late endosomes [27]. Indeed, live-cell imaging analyses revealed that in wild type, RabF2b-positive endosomes were uniformly distributed at the cell cortex; however, in the *rhd3* mutant, we found partial partitioning of endosomes into clusters (Supplementary Figure S3B). The clusters were found also in the *rhd3* mutant expressing the EE/TGN marker (AGD5) [28–30] (Supplementary Figure S4, panel A). To address whether the ER membrane clusters coincide with both EE/TGN and MVB/LE clusters (Supplementary Figure S4, panel B), we transiently overexpressed YFP-RHD3. This is known to induce aberrant ER sheets/clusters [31]. Indeed, cell imaging analyses revealed an overlap between the ER and EE/TGN/MVB/LE clusters (Supplementary Figure S4). To test whether the streaming of endosomes was compromised in *rhd3* compared with wild type, we tracked endosomes in time-lapse imaging analyses in wild type and *rhd3* leaf cotyledon cells expressing YFP-RabF2b (Figure 4c). The analyses revealed that in wild-type cells, the average velocity of RabF2b-positive endosomes (*N*_endosomes_=221) was 2.554±0.139 μm s^−1^, which is significantly higher than that in *rhd3* cells (0.373±0.039 μm s^−1^; *P<*0.0001; Figures 4d and e). These results indicate that disruption of the ER morphology due to the loss of RHD3 leads to defects in the spatial distribution and streaming velocity of endosomes. The demonstrated disruption of the dynamics of endosomes in cells that either overexpress RTNLB3 (Figure 3) or lack *RHD3* (Figure 4) argues that the observed defects in endosome distribution and dynamics are attributable to general ER network defects, and supports that ER network integrity is required for optimal streaming of endosomal organelles in plant cells.

### ER morphology defects partially disrupt endocytosis

**Figure 5:**
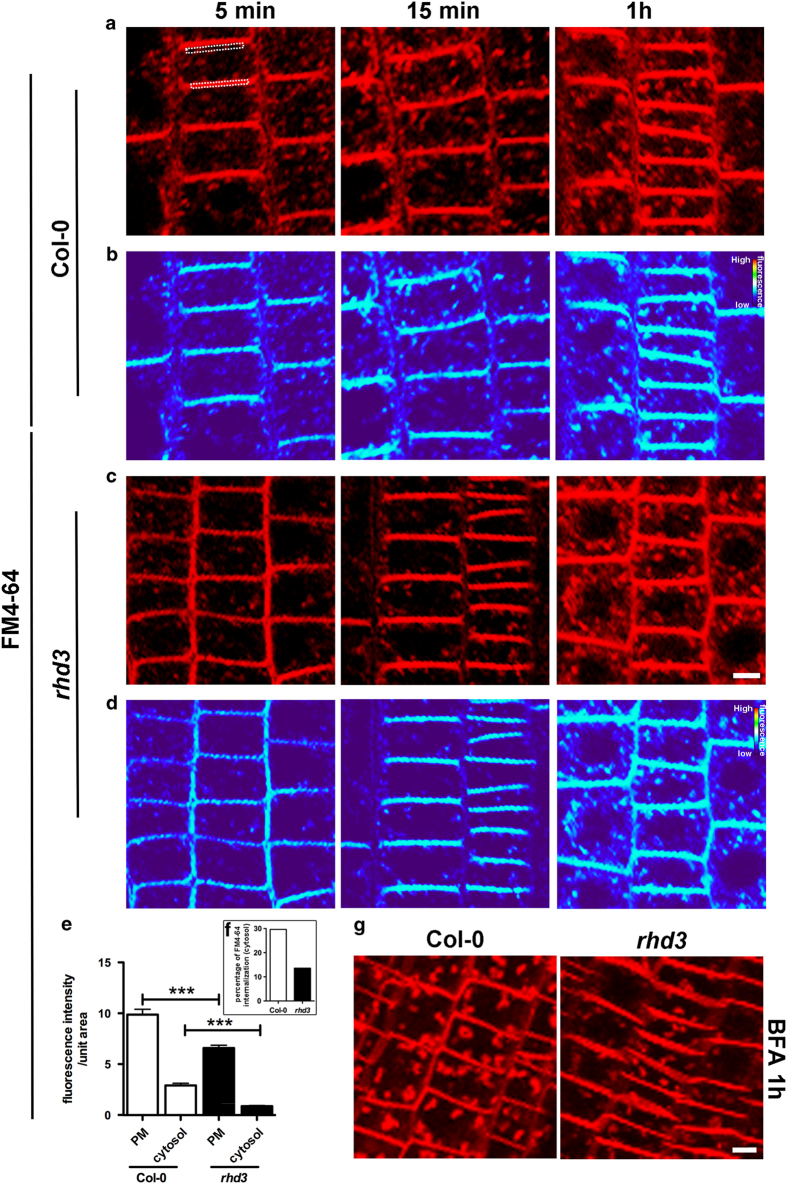
Endocytosis is delayed in *rhd3*. (**a**, **c**) Time course (5 min, 15 min, 1 h) to follow uptake of the endocytic tracer FM4-64 in Col-0 and *rhd3* seedlings. (**b**, **d**) Fluorescence signal intensities represented as heat map facilitates visualization of levels of FM4-64 uptake in the two genetic backgrounds. (**e**) The graph shows a quantification of the FM4-64 signals in the root epidermis (fluorescence signal, after 5 min of FM4-64 treatment, was measured at plasma membrane and cytosol as indicated by the white dashed rectangles in **a**). Statistical analysis was performed using Student’s *t*-test (***P<0.0001). (**f**) The graph represents the percentage of FM4-64 internalized looking at the cytosolic area. (**g**) BFA bodies observed in the maximal projection of confocal images of 6-day-old Arabidopsis seedlings (Col-0 and *rhd3* root epidermal cells) pulsed with FM4-64 marker and treated with 100 μM BFA for 1 h. BFA, brefeldin A.

We next addressed the question whether spatiotemporal anomalies in the distribution of endosomes could have an impact on cell physiology by analyzing endocytosis, which relies on endosomal function. As customary to study endocytosis homeostasis, we monitored the internalization of the endocytic tracer FM4-64 [4, 32–36] in a time-course experiment in wild type and *rhd3* root cells. Consistent with the previous observations in wild-type cells [37], FM4-64 fluorescence appeared at the PM and endosomal structures within 5 min of incubation and fluorescence internalization progressed over time (Figure 5a). However, compared with wild type, in *rhd3* cells, the internalization of the dye resulted delayed. To quantify this phenomenon, we estimated the ratio between the FM4-64 fluorescence signal at the PM and cytoplasm [38]. We found that the PM/cytoplasm ratio was significantly lower in *rhd3* compared with wild type, supporting a shift of fluorescence accumulation at the PM in *rhd3* compared with the wild-type control. These results support the possibility that endocytosis rate is reduced in *rhd3* compared with wild type (Figures 5a–f). To validate this observation, we used brefeldin A (BFA), a fungal toxin that inhibits exocytic traffic from the endosomes to the PM but does not inhibit endocytic traffic from the PM to endosomes. In wild-type cells, BFA treatment generally leads to endosome aggregations, which are known as BFA bodies, therefore disruption of endocytosis homeostasis delays formation of such bodies [39]. Consistent with this notion and the preponderant accumulation of FM4-64 at the *rhd3* PM compared with wild type (Figures 5a–f), BFA treatment led to a conspicuous appearance of BFA bodies in wild type but not in *rhd3* (Figure 5g and Supplementary Figure S5). To test whether the loss of RHD3 affected secretion, we monitored the distribution of a bulk-flow marker (SEC-RFP [40]) and a vacuolar marker (AFVY-RFP [41]) in wild type (Supplementary Figures S6A and B) and *rhd3* cells during cell expansion (Supplementary Figures S6C and D). Changes in the export ability of the ER in the mutant would be translated into variations in the subcellular distribution of these markers compared with wild type; however, no substantial differences in the distribution of the markers were noted. Together, these results show that ER defects linked to abnormal availability of the ER-shaping protein such as *RHD3* reduce endocytosis.

### Cellular availability of the auxin transport machinery is reduced in *rhd3* compared with wild type

**Figure 6:**
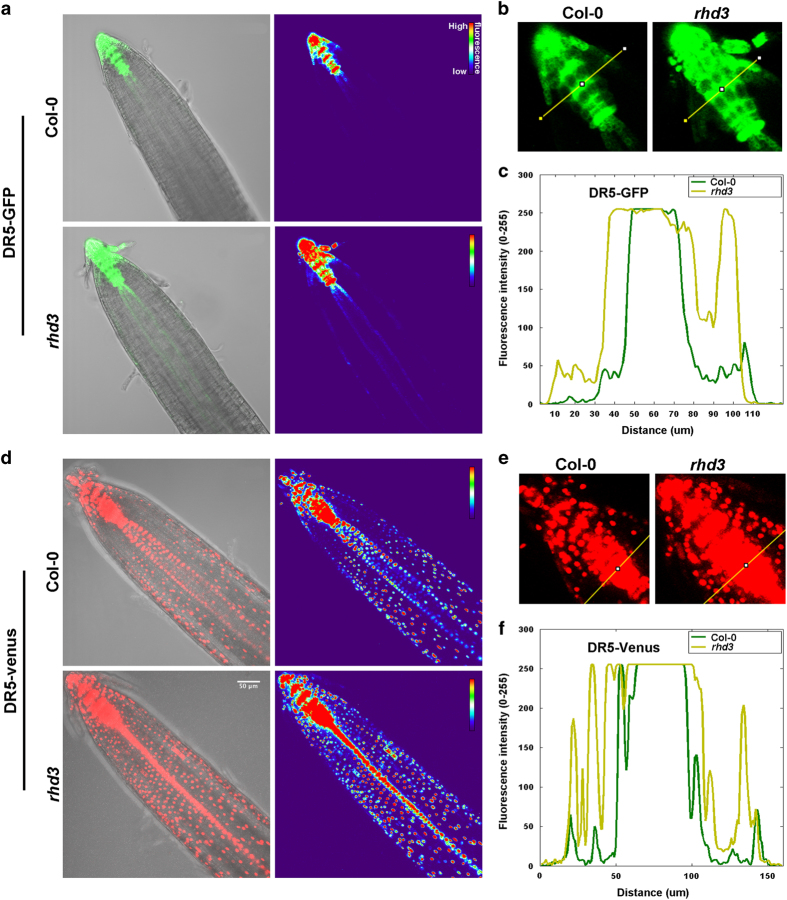
Auxin abundance. (**a**, **d**) Representative confocal image of DR5-GFP and DR5-VENUS (GFP or VENUS driven by the synthetic auxin-response promoter DR5 that reflects relative auxin levels) roots (7DAG) in wild type (Col-0) and *rhd3* showing an enlarged area with levels of auxin in the longitudinal axis *rhd3* roots compared with wild type. (**b**) Confocal image of DR5-GFP root at the apex and (**c**) quantification of the fluorescence in the graph. (**e**) Confocal image of DR5-Venus root at the apex and (**f**) quantification of the fluorescence in the graph. The images displayed are representative of at least three independent experiments with >10 seedlings examined each experiment. Scale bar, 50 μm.

**Figure 7:**
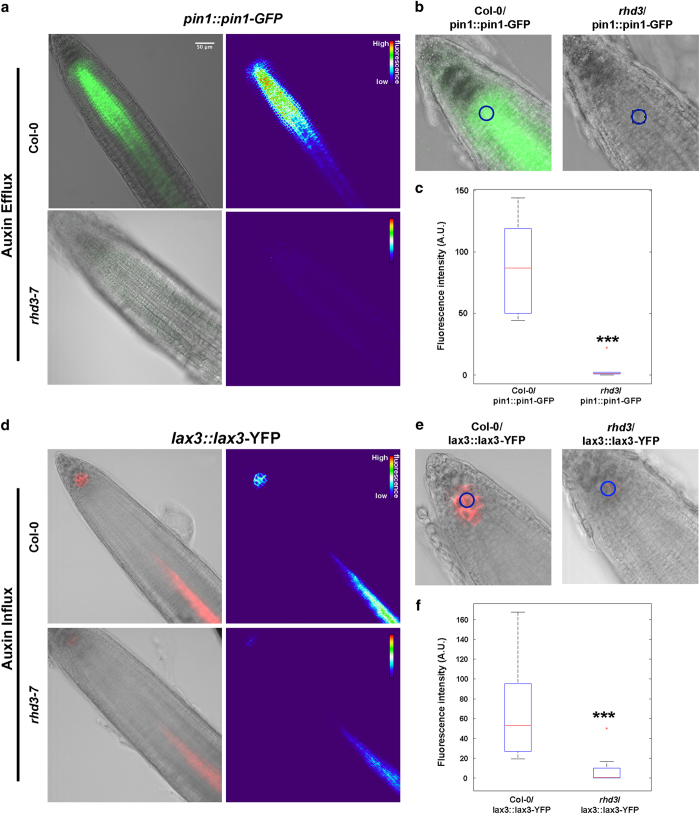
The levels of the efflux and influx machinery are compromised in *rhd3*. (**a**) Increased endogenous levels of auxin lead to a shut down of the PIN1-GFP expression in *rhd3* root compared with wild type (Col-0). (**b**). Close-up view of *PIN1::PIN1-GFP* fluorescence at the root apex shows a signal intensity differences between Col-0 and *rhd3*. (**c**) *PIN1::PIN1-GFP* signal changes can be observed in the graph (*n*=8). (**d**) L*AX3::LAX3-YFP* levels in Col-0 and *rhd3* root. (**e**) Close-up view of *LAX3::LAX3-YFP* fluorescence at the root apex shows signal intensity differences between Col-0 and *rhd3*. (**f**) LAX3-YFP signal changes can be observed in the graph (*n*=12). Statistical analysis was performed using Student’s *t*-test (***P<0.0001). Scale bar, 50 μm.

We next aimed to probe the physiological consequences of reduced levels of endocytosis in *rhd3*. In plant cells, endocytosis is critical for the intracellular trafficking and polarity of the transporters of the hormone auxin, a master regulator of plant growth [42, 43]. Application of exogenous auxin disrupts endocytosis, as demonstrated by a decrease in FM4-64 uptake in roots treated with auxin [39]. Furthermore, compared with wild type, *yucca* mutants, which are defective in auxin biosynthesis, are characterized by higher concentrations of endogenous auxin (IAA) and reduced FM4-64 internalization [39]. It is also known that high concentrations of auxin inhibit root growth [44]. Because *rhd3* mutant shows both defect in endocytosis (Figure 5) and root elongation [23, 45], we hypothesized that endogenous levels of auxin and homeostasis of auxin transport machinery would be compromised in this mutant. To test this hypothesis, we first applied IAA on wild type and *rhd3*, and established that primary root growth was inhibited in both backgrounds compared with the respective controls (Supplementary Figure S7). Intriguingly, the root growth of untreated *rhd3* seedlings was comparable to that of IAA-treated wild-type seedlings and exogenous IAA treatment led to lower root growth inhibition in *rhd3* compared with wild type (Supplementary Figure S7). These results indicated that *rhd3* is sensitive to auxin but also supported the possibility that *rhd3* roots contain higher levels of endogenous auxin compared with wild-type roots. We tested this by analyzing the fluorescence intensity of a live-auxin reporter based on the fusion of an auxin-responsive promoter DR5 to a fluorescent protein [46, 47], which enables to assess auxin levels in wild-type and *rhd3* roots. Quantitative confocal microscopy analyses of the fluorescence of the auxin reporters in the root apex indicated that the auxin levels were significantly higher in *rhd3* compared with wild type (Figure 6). These results support our initial hypothesis that the levels of auxin are compromised in *rhd3*. Because application of exogenous auxin reduces the intracellular levels of PIN-auxin transporters [48], and because we found higher auxin levels in *rhd3* compared with wild type (Figure 6), we next aimed to test whether the auxin transport machinery could be affected in *rhd3* roots. To test this, we analyzed the abundance of established fluorescent reporters of components of the auxin efflux (PIN1, PIN5) and influx (LAX3, AUX1) machinery in *rhd3* and wild-type roots. Quantitative confocal microscopy analyses showed that the fluorescence levels in transgenic lines expressing either *Pro*_*pin1*_::*PIN1-GFP* [46] (Figures 7a–c) or *Pro*_*lax3*_*::LAX3-YFP* [49] (Figures 7d–f) were significantly reduced compared with wild type. An overall decrease in the fluorescence levels could be also observed for the auxin transporters *35S::PIN5-GFP* [50] or *35S::AUX1-YFP* [51] (Supplementary Figure S8). Together, these results indicate that loss of the ER shaping RHD3 leads to increased levels of auxin and disruption of the homeostasis of transport machinery, which consequently perturbs the homeostatic distribution of these proteins at the plasma membrane.

## Discussion

Structural integrity of organelles is a requirement for their optimal function. Indeed, ER architecture defects lead to severe subcellular phenotypes, which in plants and mammals have been associated to severe growth and developmental phenotypes [14, 52–54], but the underlying mechanisms are unknown. Recent literature, however, points toward a new paradigm that disruption of the morphological integrity of the ER also affects the positioning of other organelles both in mammalian and plant cells [14, 55]. Nonetheless, the extent to which disruption of the ER network homeostasis influences the function of other organelles is yet to be defined. Our results support that ER network integrity is a requirement for the positioning and dynamics of endosomes and for endocytosis. These results not only provide evidence that association of endosomes and ER is conserved in plant and mammalian cells, they also demonstrate that endocytosis depends on ER network integrity. We propose that disruption of the ER tubular network affects the homeostasis of the subcellular distribution and dynamics of ER-associated endosomes, which is required for efficient endocytosis. By demonstrating that loss of ER structure leads also to disruption of physiological levels of auxin transporters and aberrant intracellular accumulation of auxin, we propose that ER-induced defects in endocytosis may contribute to plant growth phenotypes of mutants of the ER-shaping protein RHD3.

### The association of the ER with endosomes is conserved in eukaryotes

The ER is a pervasive organelle and arguably the organelle with the largest membrane area. Recent studies in mammalian cells have provided evidence for the existence of contact sites between endosomal organelles and ER membranes with a closer association between ER and late endosomes compared with the early endosomes [7]. Molecular candidates responsible for the connection between late endosomes and ER, like STARD3, STARD3NL and VAP, have been recently reported [56]. The identification of these proteins and of possible molecular bridges involving ORP1L-VAP (oxysterol-binding protein-VAMP-associated protein) and EGFR-PTP1B (epidermal growth factor receptor-protein tyrosine phosphatase) [57] supports that the association of the ER with endosomes may most likely exert specific functions. In this work, we have demonstrated that the ER is in extensive association with endosomes over time in plant cells. In marked contrast with mammalian cells, in plant cells, the ER is in close association with Golgi stacks, denoting a unique organization of the plant early secretory pathway. The evidence provided in this work that in plant cells endosomes are in association with the ER, similar to mammalian cells, poses that, differently from the organization of the Golgi and the ER, there is conservation of the association of the ER with post-Golgi organelles in eukaryotes.

### Endosomal streaming depends on ER integrity

It has been recently shown that defects in plant ER structure and membrane composition influence the streaming of other organelles [58]. Specifically, lack of RHD3 correlates with reduced streaming of the ER, Golgi, peroxisomes and mitochondria, independently from actin cytoskeleton integrity [14]. These findings delineated a model wherein the ER exerts a force on the movement of other organelles that are in contact with the ER [14, 59]. In this work, we have demonstrated that loss of RHD3 has a profound effect also on the streaming of endosomes. The findings that disruption of ER network homeostasis due to overexpression of RTNLB3, an ER-shaping reticulon, has a similar effect on endosome streaming compared with loss of *RHD3* argues that the observed effect is not specific to RHD3 or RTNLB3 but rather to a general loss of ER network integrity.

In this work, through photobleaching experiments we have established that overexpression of reticulon leads to changes in the ER membrane protein diffusion, as established by reduction of the mobility of a bulk ER membrane marker in conditions of RTNLB3 overexpression and RHD3 loss compared with wild type. It is possible that overabundance of reticulon may impede the homeostasis of distribution of other ER membrane proteins, which could explain alteration in the fluorescence recovery after photobleaching of a bulk membrane marker. It was similarly hypothesized that loss of RHD3 may alter the distribution of reticulons or other ER-shaping proteins and therefore change the overall ER membrane protein diffusion [14]. We speculate that if ER–endosome contacts are maintained by membrane-anchored protein tethers, the observed changes in endosome movement in the *rhd3* loss-of-function mutant as well as reticulon-overexpressing cells could be due, at least partially, to altered diffusion of the ER–endosome tethers in the ER membrane. Although this hypothesis waits for experimental validation, the evidence that membrane protein tethers between the ER and other organelles exist in non-plant organisms [59] lends partial support to our hypothesis.

It has been shown that although the ER is a contributing force to organelle streaming in plant cells, the streaming velocity can be markedly different among the various organelle types [14]. In this work, we have observed that the streaming velocity of RabF2a-positive endosomes (1.228±0.105 μm s^−1^) was significantly lower than that of RabA1g-positive endosomes (3.356±0.234 μm s^−1^) in cells with intact ER. The evidence provided in our work that the distribution of the two Rab markers do not completely overlap supports marked diversification of the streaming requirements for the various endosomal populations in plant cells. The observation that loss of ER integrity halves the average velocity of these populations of organelles supports that, although the ER is a common driving force for the streaming of both populations, the residual motor force may be exerted by diverse motor proteins that contribute to the organelle streaming with different propelling forces.

### Defects in endosome distribution due to ER network disruption compromise endocytosis, auxin homeostasis and organ growth

Live-cell imaging analyses in mammalian cells have shown that disruption of ER structure and tubule movement due to overexpression of human Reticulon 4a (Rtn4a) reduces the rate of endosome fission [55], supporting that the ER has a role in the homeostasis of endosome biogenesis. However, the consequences of loss of endosomal homeostasis due to disruption of ER integrity in the physiology of cells and of the whole organism are yet unknown. In plant cells, endosomes are highly mobile, as demonstrated earlier [6] and in this work, but it is yet to be established whether their movement has any functional relevance for the cell and organism. In our live-cell imaging analyses with the endocytic tracer FM4-64 we have found that alteration of the distribution and streaming of the endosomes is associated with a delay in FM4-64 uptake, which indicates that endocytosis is partially compromised. These conclusions are further supported by the evidence that the plasma membrane accumulation of auxin transporters, which depends on endocytosis [60], is altered in the *rhd3* mutant with defects in ER morphology. These results pose that a dysregulation in the streaming of the endosomes contribute to a dysfunction in their normal activities. The underlying causes of this phenotype are yet unknown. Endocytic and secretory cargo pass through the TGN/EE and MVBs/LEs; the latter endosome type may contain PM proteins destined to degradation and may originate from TGN/EE through maturation processes [3, 61]. Although it is possible that defects in the ER network may impede endosome maturation, it is also possible that they may compromise endosome fission and therefore reduce the number of available endosomes for endocytosis homeostasis. However, we cannot exclude that in the ER-defective mutants, the auxin defects may be also partially due to a hampered distribution of ER-localized auxin transporters due to defects in ER linked to RHD3 loss of function.

Plant growth and development depends on hormone homeostasis [43]. Small plant stature with marked reduction of root growth is a striking phenotype of all the *RHD3* mutants characterized to date [23–25]. Defects in auxin transport due to altered endocytosis are known to alter cellular auxin homeostasis [62, 63]. Furthermore, increase in auxin levels leads to a decrease in PIN protein abundance [48] and reduction in root elongation [64]. The evidence provided in our work that in the *rhd3* loss-of-function mutant the subcellular distribution of critical components of the auxin transport machinery is compromised and that the cellular levels of auxin are more elevated compared with wild type are consistent with the hypothesis of a genetic interaction of RHD3 with the auxin pathway [65]. Because *rhd3* shows severe defect in ER morphology and endosome dynamics, we propose that the association of endosomes with the ER is critical for homeostasis of endocytosis and consequently, for growth regulation in plants.

## Materials and Methods

### Cloning, plant materials and growth conditions

RTNLB3 (*AT1G64090*), RabF2a (*AT5G45130*) and RabA1g (*AT3G15060*) were cloned into either pVKH18En6 (RTNLB3) or pEarlyGateway104 (Rabs) plant expression vectors. Four-week-old *Nicotiana tabacum* (cv Petit Havana) greenhouse plants grown at 25 °C were used for *Agrobacterium tumefaciens* (strain GV3101)-mediated transient expression [66]. The bacterial optical density (OD_600_) used for plant transformation was 0.05 for tagged versions of GFP-HDEL, CNX, RTNLB3, RabF2a, RabA1g and CFP-AGD5 [15, 66], while for YFP-RHD3 it was 0.3. Homozygous transgenic *Arabidopsis thaliana* lines (Col-0 ecotype) expressing ER-YK were described previously [14]. DR5-GFP, DR5-Venus, Pin1::PIN1-GFP, LAX3::LAX3-YFP, 35S::PIN5-GFP, 35S::Aux1-YFP seeds were described earlier [47–50, 67, 68]. The transgenes were introgressed into the *rhd3* background through crosses to enable quantitative comparisons of the fluorescence intensity between wild type and *rhd3*. For IAA treatment, we used *gom8*, a recessive missense mutation in the *At3g13870* locus (*RHD3*), which is complemented by RHD3 expression [23]. *rhd3* is a T-DNA insertion null allele of *RHD3* (*rhd3*-7) [23, 69]. Seeds were surface sterilized and germinated at 21 °C under 16 h light/8 h dark conditions.

### Confocal laser scanning microscopy and image analyses

An inverted laser scanning confocal microscope Zeiss LSM510 META (http://www.zeiss.com/) was used for confocal analyses. The fluorescent proteins used in this work were GFP5 [70], Venus and EYFP. The markers used in this study were the ER lumenal marker, ER-YK [71], and the ER membrane markers: RTNLB3 and calnexin [72], fused to GFP. RabF2a, RabF2b and RabA1g tagged with fluorescent proteins were used as endosome markers [27, 35]. Imaging settings for single fluorochromes or combinations were as described previously [40, 73, 74]. To analyze the velocity of the endosomes, 23 time-lapse sequences for RabF2a and RabA1g (50 frames per time-lapse sequence), and 43 time-lapse sequences for RabF2b (100 frames for each time-lapse sequence) were recorded in the cortical region of cotyledon pavement cells. The time-lapse frames (512×512 pixel or 256×256 resolution) were captured with 2 μm pinhole diameter, 10% power of an argon 488 nm laser line, and four digital zoom using an EC Plan-Neofluar ×40/1.30 oil M27 objective. Kimograph analysis was performed in post acquisition using ImageJ plugins (v1.49). Velocity values were calculated by averaging the velocity of each endosome in each time-lapse sequence; then mean values were calculated as the mean of the velocities estimated in the 23 or 43 time-lapse sequences. Velocity values were estimated by averaging data values of each time-lapse sequence for each sample. Statistical analysis was based on *t*-test for velocity measurement and for FRAP data. Photoshop (http://www.adobe.com/) was used for further image handling.

### FRAP analyses

Leaf segments were incubated in latrunculin B (25 μm), which is a potent disrupter of actin filaments [75], for 30 min before imaging, as described earlier [15]. For FRAP experiments, the ER in the cortical region of abaxial epidermal pavement cells of *N. tabacum* leaves was used. Ten prebleach scans were captured using settings for GFP with the 488-nm laser transmission set to 1–2% transmission before bleaching of a 3.6 μm^2^ spot using 30 iteractions of a 488/514-nm laser lines set to 100% transmission. The number of bleach events captured was 66 for CNX-GFP, and 68 for GFP-RTNLB3. Normalized data were plotted using MatLab (www.mathworks.com), as described previously [28].

### Drug and dye treatments

Six-day-old Arabidopsis seedlings were incubated in 0.5× MS medium with 2 μM FM4-64 (500 μM; stock DMSO) for 5 min, washed three times at room temperature, mounted, and observed. For BFA treatment, the seedlings were first incubated for 5 min with 2 μM FM4-64 then incubated 1 h in 100 μM BFA solution. Confocal microscopy analyses of the FM4-64-stained seedlings were performed on epidermal cells of the root tip.

### Electron microscopy

Root tips from 1-week-old Arabidopsis seedlings were high-pressure frozen in a Baltec HP010, freeze-substituted in 2% OsO_4_ in acetone, and embedded in Epon resin as described previously [76]. For electron tomography, tilt series from 300-nm-thick sections were collected between −60º and +60º, every 1° in a FEI Tecnai F30 electron microscope operating at 300 kV. Tomogram calculation and segmentation was done with the IMOD package [77].
