# Supplementary figures and images for: ER network homeostasis is critical for plant endosome streaming and endocytosis
Source: Cell Discov. 2015 Nov 17;1:15033–. doi: 10.1038/celldisc.2015.33 (PMC4860783; doi:10.1038/celldisc.2015.33)

**A**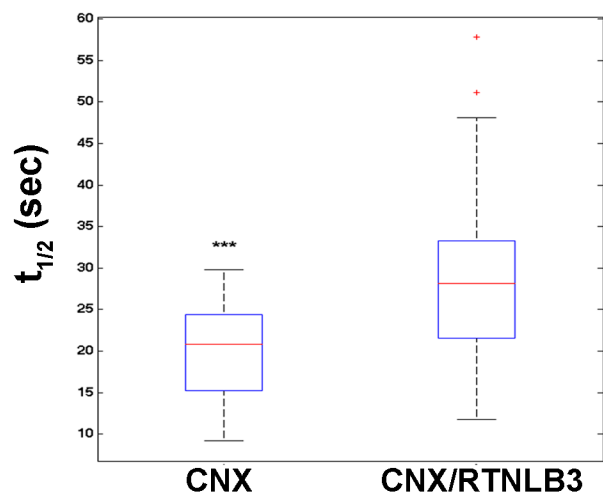**B**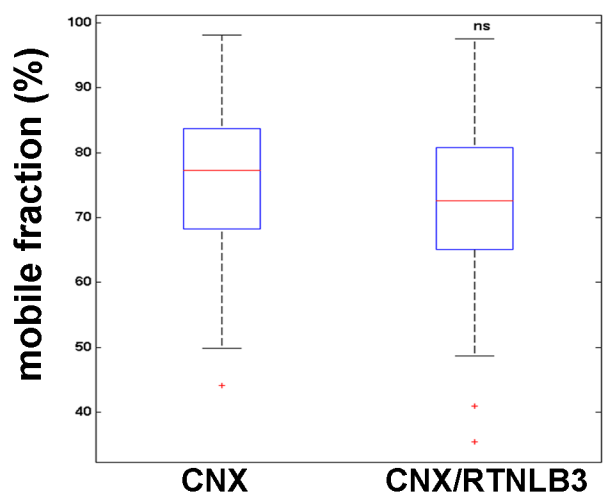**Figure S1**

Supplement: Supplementary Figure S1 [file celldisc201533-s1.pdf]

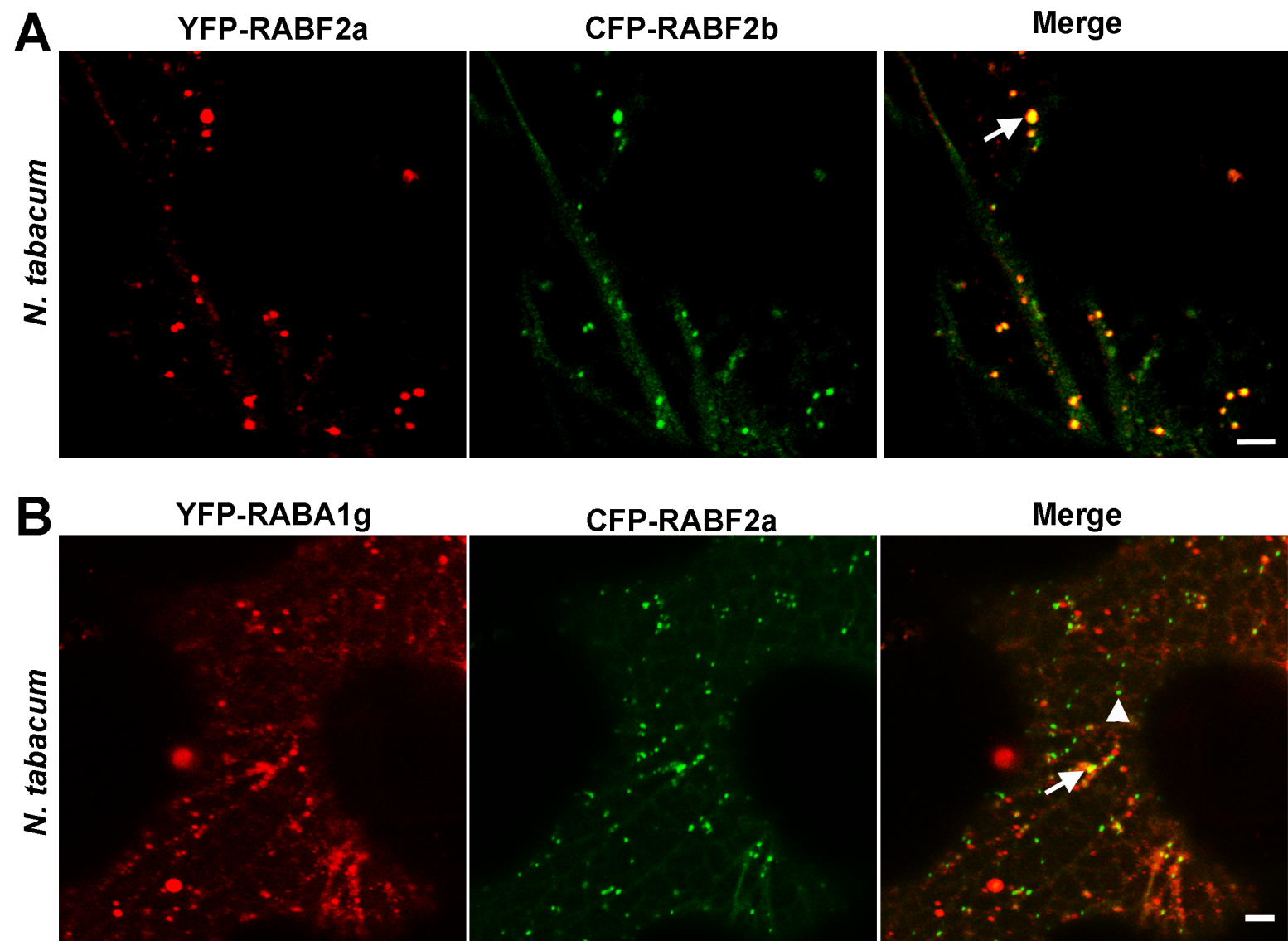

Figure S2

Supplement: Supplementary Figure S2 [file celldisc201533-s2.pdf]

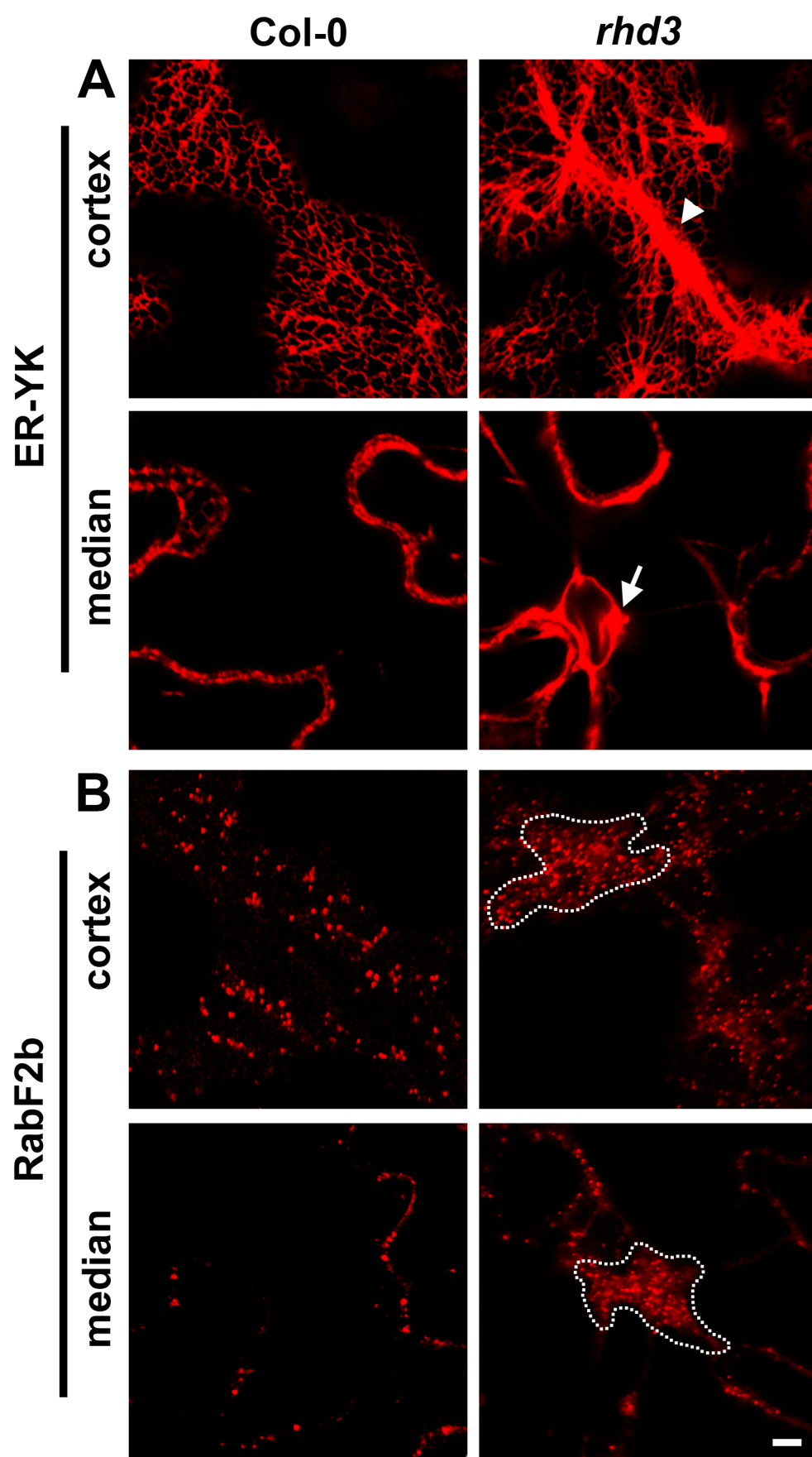

Figure S3

Supplement: Supplementary Figure S3 [file celldisc201533-s3.pdf]

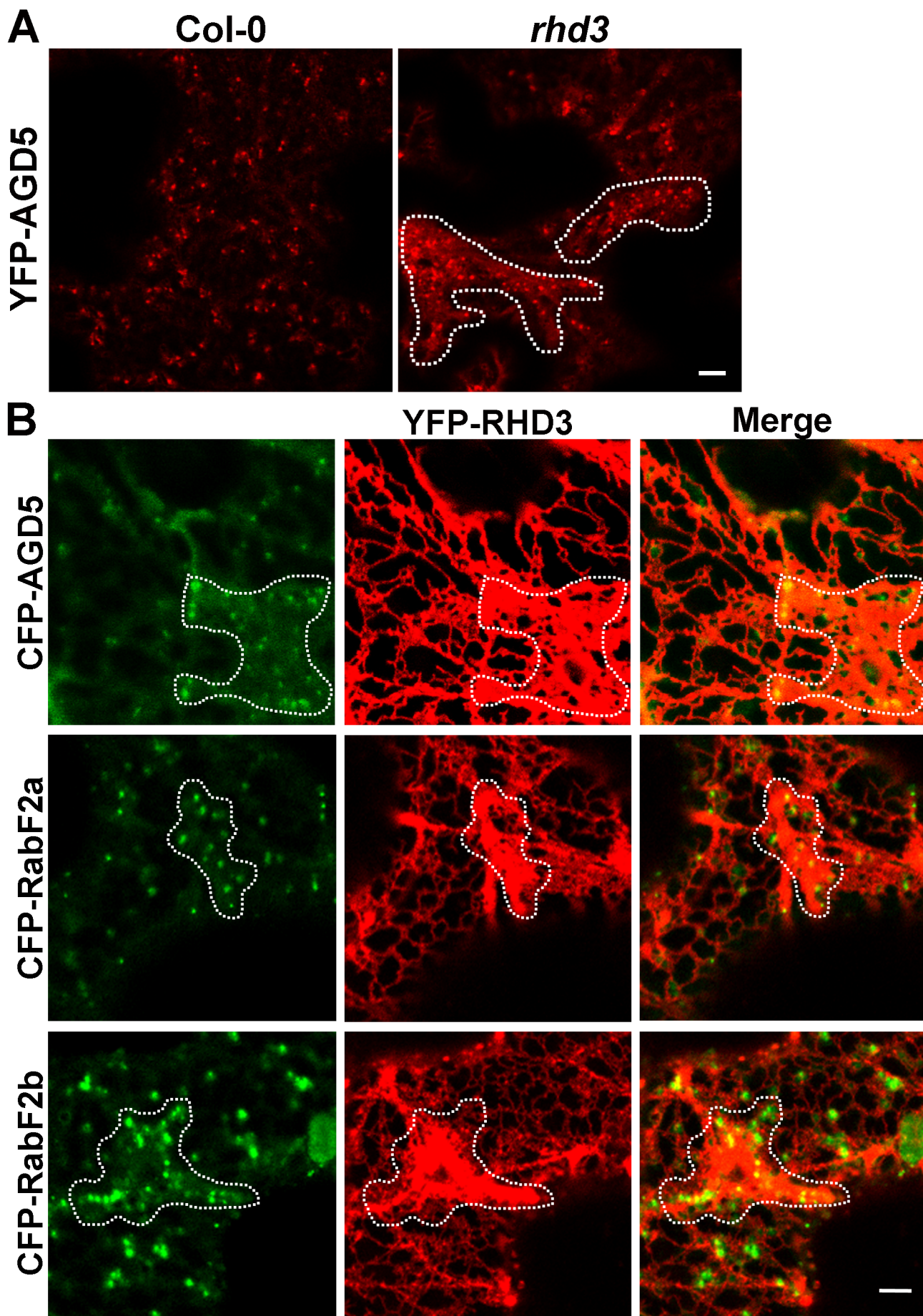

Figure S4

Supplement: Supplementary Figure S4 [file celldisc201533-s4.pdf]

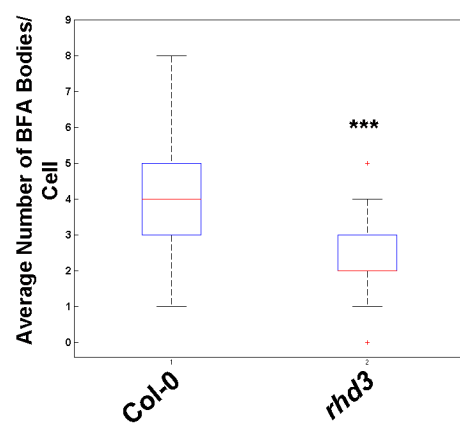

**Figure S5**

Supplement: Supplementary Figure S5 [file celldisc201533-s5.pdf]

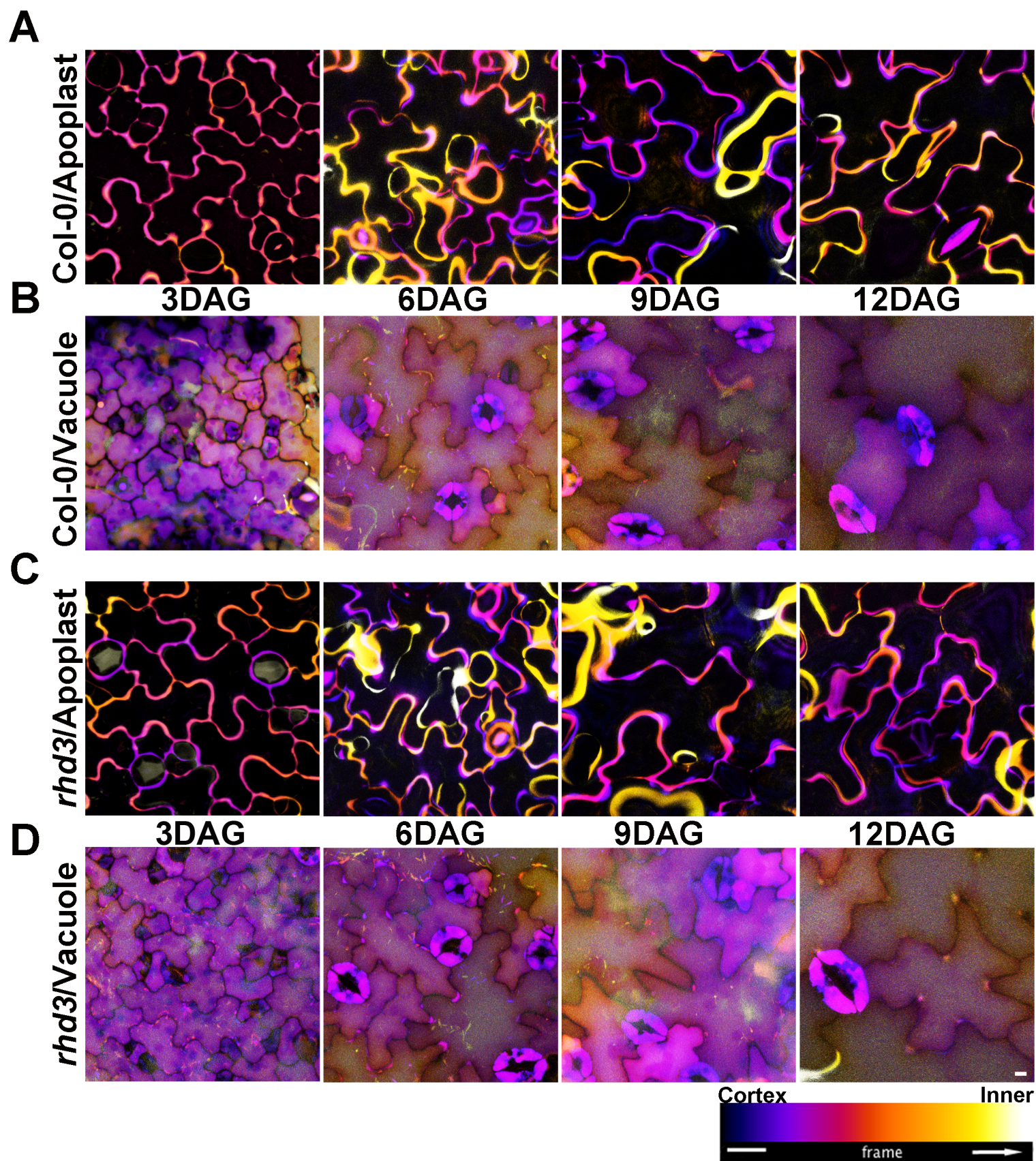

Figure S6

Supplement: Supplementary Figure S6 [file celldisc201533-s6.pdf]

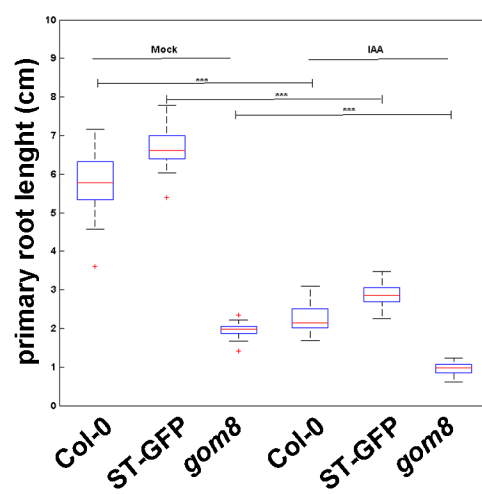

Figure S7

Supplement: Supplementary Figure S7 [file celldisc201533-s7.pdf]
