# Supplementary material for: ER network homeostasis is critical for plant endosome streaming and endocytosis
Source: Cell Discov. 2015 Nov 17;1:15033–. doi: 10.1038/celldisc.2015.33 (PMC4860783; doi:10.1038/celldisc.2015.33)
Supplement: Supplementary Information [file celldisc201533-s10.doc]

**Supplementary Figure S1. A-B. Overexpression of GFP-RTNLB3 reduces membrane protein diffusion in the ER membrane.** ER membrane protein diffusion measurements using fluorescence recovery after photobleaching (FRAP) analysis in cells expressing the ER bulk membrane marker calnexin-GFP (calnexin transmembrane domain and cytosolic tail fused to GFP; CNX-GFP) alone or in combination with YFP-RTNLB3 revealed that overexpression of the reticulon fusion reduces the motility of CNX-GFP, compared to cells expressing CNX-GFP alone. Analysis of FRAP experiments showing the half time (**A**) and mobile fraction (**B**) in tobacco epidermal cells expressing the ER membrane marker calnexin–GFP (CNX–GFP) alone or with RTNLB3-GFP. Statistical analysis was performed using Student’s t-test (***P<0.0001).

**Supplementary Figure S2. Co-expression analyses indicate partial overlap of endosome markers. (A)** Confocal images of epidermal cells (*N. tabacum*) coexpressing the endosomes marker YFP-RabF2b and CFP-RabF2a. **(B)** Confocal images of epidermal cells (*N. tabacum*) co-expressing the endosomes marker CFP-RabF2a and YFP-RabA1g. Scale bars = 5 μm.

**Supplementary Figure S3. ER network influences spatial distribution of endosomes.** **A.** Confocal images of the ER in the cortical and median regions of *A. thaliana* cotyledon leaf epidermal cells in wild type (Col-0; left panel) and *rhd3* (right panel). The ER is visualized with the ER lumen marker ER-YK. Note the elongated ER tubules in *rhd3* (arrowhead). Arrow points to the nuclear envelope. **B.** Confocal images of late endosomes/pre‐vacuolar compartments labeled with YFP-RabF2b in *A. thaliana* cotyledon epidermal cells in wild-type (Col-0; left panel) and *rhd3* (right panel). Images were acquired in the cortical and median regions of the cells. Note the clustering of endosomes in *rhd3* (circled by dotted line). Scale bars = 5 μm.

**Supplementary Figure S4. Spatial overlapping of ER and endosome clusters. A.** Confocal images of EE/TGN compartments labeled with YFP-AGD5 in *A. thaliana* cotyledon epidermal cells in wild type (Col-0; left panel) and *rhd3* (right panel). Images were acquired in the cortical region of the cells. Note the clustering of EE/TGN organelles in *rhd3* (circled by dotted line). **B.**Confocal microscopy analyses of *N. tabacum* epidermal cell expressing YFP-RHD3 show that at a high optical density of agrobacterium (OD600 = 0.3) aberrant ER sheets/clusters are induced, in agreement with earlier reports[^31^](#_ENREF_31). Note that the ER clusters are associated with clusters of EE/TGNs and MVB/LEs (clusters are circled by dotted line). Scale bars = 5 μm.

**Supplementary Figure S5. BFA bodies number.** The average number of BFA bodies of 6-day-old Arabidopsis seedlings pulsed with FM4-64 and treated with 100 μM BFA for 1 h is displayed as number of BFA bodies observed in the maximal projection of confocal images per cell. Statistical analysis was performed using Student’s t-test (***P<0.0001).

**Supplementary Figure S6.** **Distribution of apoplast and vacuolar markers.** (A, C) Distribution of apoplast and vacuole markers in Col-0 and *rhd3* cells. Z-stack projections of apoplast marker SEC-RFP and the vacuole marker AFVY-RFP in wild-type background. (B, D) Z-stack projection of SEC-RFP and AFVY-RFP in *rhd3*. The max projection analysis shows a normal distribution of the markers also in mutant plant cells with no visible marker retention in the ER. Scale bars = 5 μm.

**Supplementary Figure S7. Primary root length in *rhd3*.** The graph shows IAA treatment on Col-0, ST-GFP and gom8 (Loss-of-function allele of ROOT HAIR DEFECTIVE3 (RHD3) seedlings to evaluate the response of the mutant in presence of exogenous auxin. For statistical analysis ANOVA was applied, with a Tukey's post-hoc test.

**Supplementary Figure S8. Pin5-GFP and Aux1-YFP levels. A.** Increase of endogenous auxin levels leads to decrease in PIN5-GFP abundance. Scale bar = 50 μm. **B.** Aux1-YFP levels in Col-0 and *rhd3* root. Reduction in the Aux1-YFP signal occurs in *rhd3* compared to wild type. The images displayed are representative of at least three independent experiments with >10 seedlings examined in each experiment. Scale bar = 50 μm.

**Supplementary Movie S1. ER and endosomes are associated in plant cells**. Time-lapse microscopy (50 frames/28.08 sec) in the cortical region of a *N. tabacum* leaf epidermal cells expressing GFP-HDEL (ER lumen) and YFP-RabF2a (late endosome/MVBs) reveals that the majority of the endosomes are associated with the ER membranes over time.
