# Supplementary material for: ER network homeostasis is critical for plant endosome streaming and endocytosis
Source: Cell Discov. 2015 Nov 17;1:15033–. doi: 10.1038/celldisc.2015.33 (PMC4860783; doi:10.1038/celldisc.2015.33)
Supplement: Supplementary Figure S8 [file celldisc201533-s8.pdf]

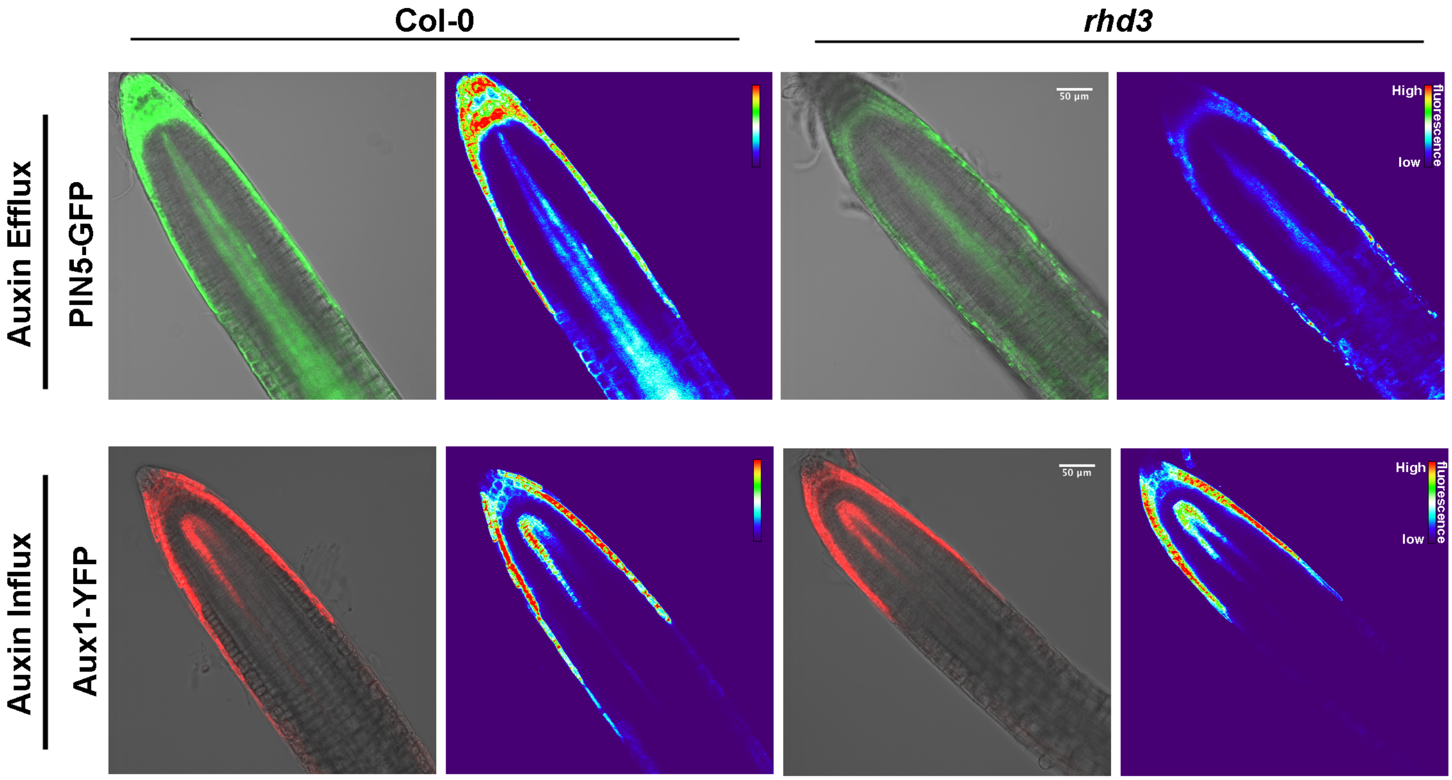

Col-0
rhd3
Auxin Efflux
PIN5-GFP
Auxin Influx
Aux1-YFP
50 µm
High
low
fluorescence

Figure S8
